# Supplementary material for: High Incidence of Inappropriate Alarms in Patients with Wearable Cardioverter-Defibrillators: Findings from the Swiss WCD Registry
Source: J Clin Med. 2021 Aug 25;10(17):3811. doi: 10.3390/jcm10173811 (PMC8432131; doi:10.3390/jcm10173811)
Supplement: Supplementary file 1 [file jcm-10-03811-s001.zip › jcm-1307381-supplementary.pdf]

Supplementary Material

Table S1. Artefacts in WCD patients.

	<3 artefacts per month (n=249)	≥3 artefacts per month (n=207)	Total (n=456)	p value
**Age**	57.5 (14.2)	56.6 (13.0)	57.3 (14.0)	0.583
Female	62 (17.1%)	19 (20.4%)	81 (17.8%)	
Male	301 (82.9%)	74 (79.6%)	375 (82.2%)	
**BMI**	26.2 (5.5)	27.1 (6.8)	26.4 (5.8)	0.187
**LVEF**	30.9 (13.2)	33.8 (12.8)	31.5 (13.1)	0.054
**Indication for WCD**				0.291
Low LVEF	126 (34.7%)	26 (28.0%)	152 (33.3%)	
Recent myocardial infarction	92 (25.3%)	33 (35.5%)	125 (27.4%)	
Bridging*	53 (14.6%)	16 (17.2%)	69 (15.1%)	
Recent PCI or CABG with low LVEF	30 (8.3%)	6 (6.5%)	36 (7.9%)	
VT with normal LVEF	29 (8.0%)	6 (6.5%)	35 (7.7%)	
Percutaneous or surgical valve repair with low EF	9 (2.5%)	4 (4.3%)	13 (2.9%)	
Syncope	5 (1.4%)	1 (1.1%)	6 (1.3%)	
Other	19 (5.2%)	1 (1.1%)	20 (4.4%)	
**Underlying heart disease**				0.218
Ischemic cardiomyopathy without coronary dissection	209 (57.6%)	64 (68.8%)	273 (59.9%)	
Non-ischemic cardiomyopathy	93 (25.6%)	15 (16.1%)	108 (23.7%)	
Valvular	19 (5.2%)	4 (4.3%)	23 (5.0%)	
Myocarditis	8 (2.2%)	2 (2.2%)	10 (2.2%)	
Channelopathy	9 (2.5%)	0 (0.0%)	9 (2.0%)	
Congenital	4 (1.1%)	3 (3.2%)	7 (1.5%)	
Ischemic cardiomyopathy with coronary dissection	2 (0.6%)	0 (0.0%)	2 (0.4%)	
Other	19 (5.2%)	5 (5.4%)	24 (5.3%)	
**Medical therapy**				
Betablocker	332 (91.5%)	82 (88.2%)	414 (90.8%)	0.328
ACEi, ARB or neprilysin inhibitor	44 (12.1%)	12 (12.9%)	56 (12.3%)	0.838
Aldosterone antagonist	223 (61.4%)	56 (60.2%)	279 (61.2%)	0.830
Amiodarone	61 (16.8%)	19 (20.4%)	80 (17.5%)	0.412
**Atrial fibrillation**	91 (25.1%)	17 (18.3%)	108 (23.7%)	0.169
**Atrial flutter**	27 (7.4%)	8 (8.6%)	35 (7.7%)	0.707
**Other supraventricular tachycardia**	352 (97.0%)	93 (100.0%)	445 (97.6%)	0.089

Data are displayed according to number of alarms emitted by the WCD per months. Mean (SD) and number (%). * Bridging until ICD-reimplantation, until primary ICD implantation or until heart transplant. Abbreviations: ACEi = angiotensin converting enzyme inhibitor; ARB = angiotensin II receptor antagonist; BMI = body mass index; CABG = coronary artery bypass graft; LVEF = left ventricular ejection fraction; PCI = percutaneous coronary intervention; VT = ventricular tachycardia.

Table S2. Outcome based on VT/VF recordings.

	No VT/VF recordings (n=435)	≥1 VT/VF recordings (n=21)	Total (n=456)	p value
**ICD implanted**				< 0.001#
ICD	194 (45.8%)	18 (85.7%)	212 (47.6%)	
None	230 (54.2%)	3 (14.3%)	233 (52.4%)	
**First treatment by ICD after implantation**				0.081
Adequate treatment	18 (9.2%)	4 (22.2%)	22 (10.3%)	
No adequate treatment	178 (90.8%)	14 (77.8%)	192 (89.7%)	
**Time to treatment after implantation (days)**	323.4 (314.2)	63.0 (104.0)	286.2 (306.4)	0.117

Data are displayed mean (SD) and as number (%). Abbreviations: ICD = implantable cardioverter-defibrillator; WCD = wearable cardioverter-defibrillator. # statistically significant results.

.

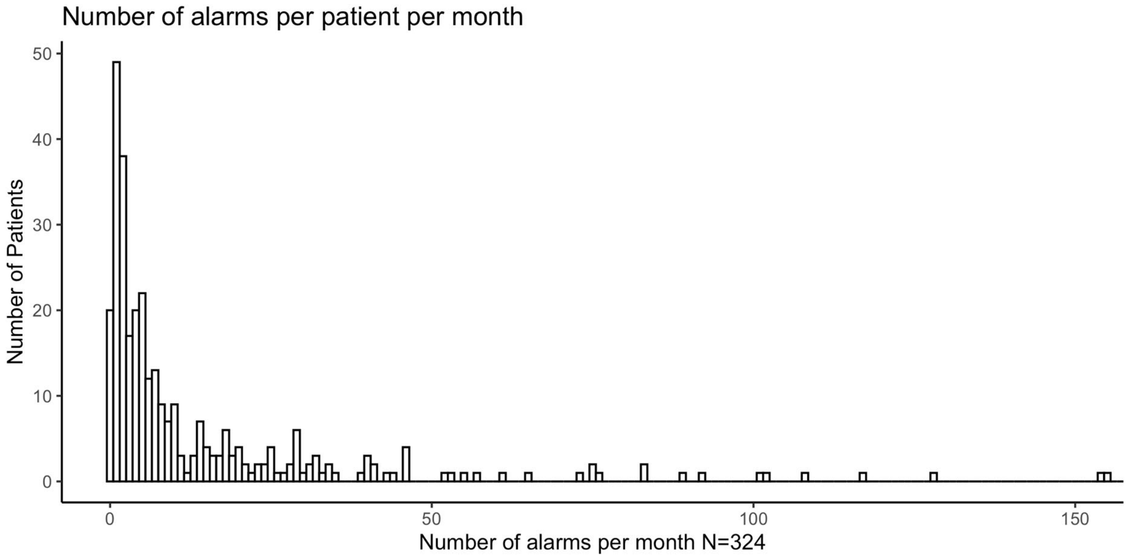

Figure S1. Distribution of alarm burden in patients with at least one alarm per month (Outliers with >150 alarms per months are not illustrated).
